# The role of natural killer T cells in liver transplantation

**DOI:** 10.3389/fcell.2023.1274361

**Published:** 2024-01-05

**Authors:** Wenchao Zhao, Mingqian Li, Shifei Song, Yao Zhi, Chen Huan, Guoyue Lv

**Affiliations:** ^1^ Department of Hepatobiliary and Pancreatic Surgery, General Surgery Center, The First Hospital of Jilin University, Changchun, Jilin, China; ^2^ Center of Infectious Diseases and Pathogen Biology, Institute of Virology and AIDS Research, Key Laboratory of Organ Regeneration and Transplantation of The Ministry of Education, The First Hospital of Jilin University, Changchun, Jilin, China

**Keywords:** NKT cells, NK-like T cells, NKT cell subpopulation, transplantation immunity, immunotherapy

## Abstract

Natural killer T cells (NKTs) are innate-like lymphocytes that are abundant in the liver and participate in liver immunity. NKT cells express both NK cell and T cell markers, modulate innate and adaptive immune responses. Type I and Type II NKT cells are classified according to the TCR usage, while they recognize lipid antigen in a non-classical major histocompatibility (MHC) molecule CD1d-restricted manner. Once activated, NKT cells can quickly produce cytokines and chemokines to negatively or positively regulate the immune responses, depending on the different NKT subsets. In liver transplantation (LTx), the immune reactions in a series of processes determine the recipients’ long-term survival, including ischemia-reperfusion injury, alloresponse, and post-transplant infection. This review provides insight into the research on NKT cells subpopulations in LTx immunity during different processes, and discusses the shortcomings of the current research on NKT cells. Additionally, the CD56-expressing T cells are recognized as a NK-like T cell population, they were also discussed during these processes.

## 1 Introduction

Natural killer T (NKT) cells are considered a type of non-traditional T cells that exhibits rapid responses against lipid antigens, restricted T cells receptors (TCRs) diversity, and share characteristics and functions of both innate immune system and adaptive immune system (Werner et al., 2011; Godfrey et al., 2015; Godfrey et al., 2018; Shimizu et al., 2020). In the process of understanding NKT cells, the definition and classification of NKT cells have been continuously revised and improved. They can be categorized into two subtypes based on the type of TCR they express: Type I NKT cells and Type II NKT cells (Godfrey et al., 2004). In addition, a CD56 expressing T cell population bears NK cell activating and inhibitory receptors on the surface, and their function are regulated through these receptors, they are referred to as “NK-like T cells” albeit they possess “CD1d-independent” antigen recognition and MHC-unrestricted capabilities (Almeida et al., 2023; Kratzmeier et al., 2023).

NKT and NK-like T cells playing crucial roles in liver pathology. In viral hepatitis, NKT cells primarily exhibit viral clearance effect by secreting IFN-γ during acute infection, while they also mediate hepatocyte injury by lysis infected cells or producing cytokines during chronic infection (Malhi and Gores, 2008; Huang W. et al., 2018). The effects of NKT cells on the development of NAFLDs are rather controversial. While NKT cells ameliorate NAFLDs, probably by improving insulin resistance, they are also likely to play a pro-inflammatory role in NAFLDs (Elinav et al., 2006; Tajiri and Shimizu, 2012). NKT cells promote the development of hepatic cell injury through three mechanisms: 1) NKT cells directly induce liver cell damage by expressing FasL and secreting TNF-α, perforin, and granzyme B; 2) NKT cell-derived IFN-γ induces the differentiation of Th0 cells into Th1 cells, enhancing their cytotoxic effects; 3) NKT cells secrete IL-4, promoting the differentiation of Th0 cells into Th2 cells and enhancing B cell production of antibodies (Santodomingo-Garzon and Swain, 2011). Additionally, TNF-α and IFN-γ are involved in the recruitment of functional T cells, while IL-4 may facilitate the infiltration of neutrophils into the liver (Mattner, 2013). NKT cells also serve as potent activators of T regulatory cells, which have a negative impact on immune responses, thereby alleviating autoimmune liver injury (Du et al., 2017). Statistical data indicate that simultaneous inhibition of NKT cells and promotion of T regulatory cells in experimental animal models contribute to the mitigation of autoimmune liver injury (Gao et al., 2019).

Liver transplantation (LTx) has become an optimal choice for end-stage liver disease. Liver acts as an innate immunity-dominant organ (Lantz and Bendelac, 1994; Fahrner et al., 2016). Among various organ systems in humans, the liver has the highest NKT-cell/T-cell ratio, with NKT cells accounting for approximately 20% of intrahepatic lymphocytes (Emoto and Kaufmann, 2003; Exley and Koziel, 2004; Nemeth et al., 2009). NKT cells play a crucial role in connecting the innate and adaptive immune systems by directly interacting with hepatocytes, macrophages (Kupffer cells), T cells, and dendritic cells through cell-to-cell contact or the secretion of cytokines (Gu et al., 2022). This review summarizes the classification and function of NKT cells in LTx, pointing out the shortcomings of some research on NKT cells subpopulations, and providing reference for its clinical application.

## 2 Classification of NKT cells

### 2.1 Type I NKT cells

Type I NKT cells, also known as invariant NKT (iNKT) cells, possess an invariant TCR-α chain rearrangement that is paired with a limited repertoire of TCR-β chains. In mice, this invariant TCR-α chain is Vα14Jα18 (TRAV11-TRAJ18), which is paired with Vβ7 (TRB29), Vβ8.2 (TRB13-2), or Vβ2 (TRBV1). Meanwhile, in humans, the invariant TCR-α chain is Vα24Jα18 (TRAV10-TRAJ18), which is paired with V-β11 (TRBV25-1) (Lantz and Bendelac, 1994) (Table 1). iNKT cells can recognize lipid antigens (such as glycosphingolipids, glycerophospholipids, lysophospholipids, and cholesterol ester) presented by CD1d, a non-polymorphic MHC class I-like molecule (Mattner et al., 2005; Bendelac et al., 2007; Kumar, 2013; Wang and Yin, 2015). Out of all the lipid antigens, α-galactosylceramide (α-GalCer, also known as KRN7000), a synthetic compound, was the first to be experimentally validated for its ability to enhance the activity of iNKT cells (Kobayashi et al., 1995; Bendelac et al., 2007; Shimizu et al., 2010). The discovery of this glycolipid was made from an extract of the marine sponge Agelas Mauritianus, and its impact on the activation of iNKT cells has been extensively documented (Godfrey et al., 2010).

**TABLE 1:** Phenotypic markers of NKT cells.

		CD1d-independent	α-GalCer reaction	TCRs	
				Human	Mice
NKT cells	iNKT	No	Yes	Vα24Jα18 (TRAV10-TRAJ18), V-β11 (TRBV25-1)	Vα14Jα18 (TRAV11-TRAJ18), Vβ7 (TRB29), Vβ8.2 (TRB13-2), Vβ2 (TRBV1)
	dNKT	No	No	Vα (TRAV7, TRAV9, TRAV12)	Diverse
NK-like T cells		Yes	No	No	

Crosby and Kronenberg (2018) have divided the mouse iNKT cells into five subgroups. They are similar to Th cell subsets, including iNKT1, iNKT2, iNKT17, iNKT10, and follicular help iNKT (iNKTfh). Human DN and CD8^+^ iNKT cells were found to exhibit similarities to mouse iNKT1 cells, displaying heightened secretion of IFN-γ and enhanced cytotoxic activity upon activation (Gumperz et al., 2002; Lee et al., 2002). Among these, iNKT2 cells secrete cytokines such as IL-4 and IL-13, which bear a resemblance to Th2 cells (Crosby and Kronenberg, 2018). They have also identified iNKT10 cells, which produce IL-10 and share similarities with type 1 regulatory T cells (Sag et al., 2014; Lynch et al., 2015; Wingender et al., 2015). The primary function of iNKT10 cells is to suppress the proliferation and activation of T cells, thereby regulating immune suppression and tolerance. Whilst the other two subgroups of iNKT cells were not explicitly mentioned in human research. Compared with other tissues with frequencies ranging from 0.1% to 0.2%, such as the thymus, blood, bone marrow, and lymph nodes, iNKT cells are mainly present in the mouse liver, accounting for 10%–30% of the total cell population. In humans, the liver contains a significantly higher number of iNKT cells compared to other tissues in the body, making up approximately 1% of the total lymphocyte population in the liver (Gao et al., 2009; Schipper et al., 2012), whereas in peripheral tissues, such as blood and lymph nodes, their frequency ranges from 0.01% to 0.5% (Watarai et al., 2008; Berzins et al., 2011). This is also the most thoroughly and comprehensively studied group of NKT cells (Wang and Yin, 2015).

### 2.2 Type II NKT cells

Type II NKT cells, also referred to as diverse NKT (dNKT) cells, do not possess the invariant TCR-α chain and instead use a variety of TCR-α chains (including TRAV7, TRAV9, and TRAV12) (Cardell et al., 1995; Macho-Fernandez and Brigl, 2015) and TCR-β chains (Treacy et al., 2014). In contrast to iNKT cells, dNKT cells have the ability to recognize self-lipids, such as lysophosphatidylcholine (LPC) and sulfatides (Maricic et al., 2014; Treacy et al., 2014; Stax et al., 2017). They are CD1d-restricted like iNKT cells, but they are unable to recognize α-linked glycolipids such as α-Galcer. However, they do react with β-linked glycolipids (Maricic et al., 2014). Hence, to differentiate between iNKT cells and dNKT cells using cytofluorography, one can utilize staining with α-GalCer/CD1d- or sulfatide/CD1d-tetramers (Jahng et al., 2004; Arrenberg et al., 2010).

Although dNKT cells are less common in mice and more challenging to study, they are predominant in humans (Arrenberg et al., 2009; Rhost et al., 2012; Kumar and Delovitch, 2014). There are subsets of dNKT cells that exhibit distinct TCR repertoires and cytokine secretion patterns in various tissues, similar to the different subsets observed in iNKT cells (Marrero et al., 2015). However, so far, there is no clear classification of subsets of dNKT cells, depending on their function, dNKT cells can be approximately classified into pro-inflammatory and anti-inflammatory subsets (Maricic et al., 2014).

## 3 Natural Killer-like T cells

In recent years, the definition of Natural Killer-like T cells is shrouded in ambiguity. The existence of NKT cell subsets and other T cell types that exhibit similarities to NKT cells has led to perplexity in the literature (Almeida et al., 2023). To establish a precise distinction between classical NKT cells and NK-like T cells, scholars have suggested raising the definition of NK-like T cells which co-express CD3^+^ and CD56^+^ and CD1d non-restricted (Almeida et al., 2023). Although there may be certain resemblances between these two populations, NK-like T cells are a distinct subset of exquisitely specialized conventional or unconventional T cells, which exhibit the expression of CD56 and other natural killer receptors, and are capable of recognizing antigens in a manner that is independent of CD1d.

In adulthood, NK-like T cells represent a small fraction of lymphocytes in circulation, amounting to under 10% (Romero-Olmedo et al., 2021). NK-like T cells exhibit the expression of natural cytotoxic receptors (NCR) such as NKp44 and NKp46, as well as activation-related markers like CD69 and HLA-DR (Chan et al., 2013; Romero-Olmedo et al., 2021). Additionally, they express the same NKR, costimulatory, chemokine, and cytokine receptors as iNKT cells (Eger et al., 2006; Chan et al., 2013). The phenotype of NK-like T cells is also constantly changing, with distinct phenotypes at naïve, memory, type 1, 2, and 17 differentiation stages (Almeida et al., 2023).

## 4 Interaction of NKT cells with other immune cells

**FIGURE 1:**
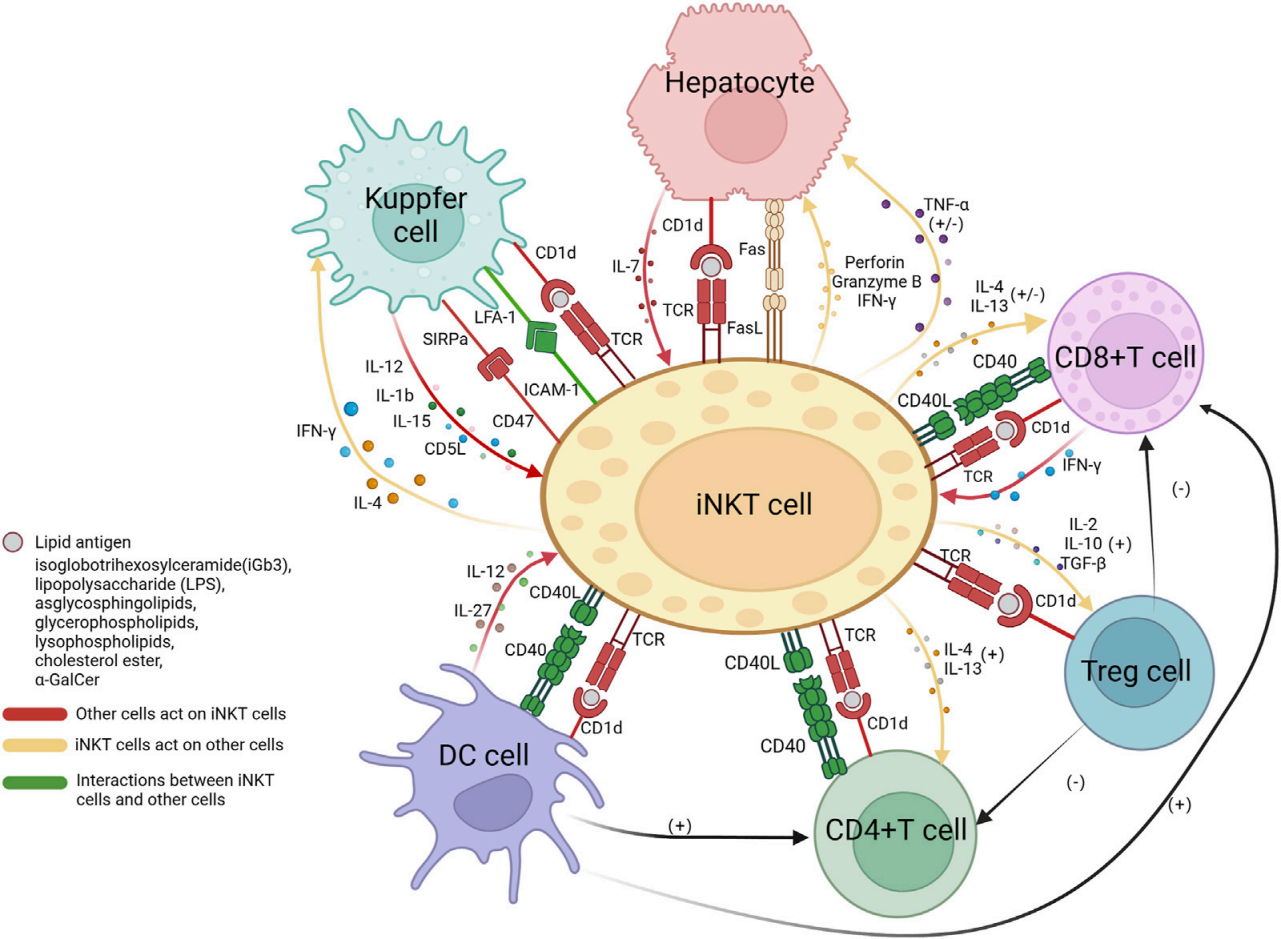
The interactions between iNKT cells and hepatocytes, CD4^+^ T cells, CD8^+^ T cells, Tregs cells, Kupffer cells, and dendritic cells. The presentation of lipid antigens to iNKT cells by all cells is dependent on the CD1d pathway, the interaction between CD40 on the surface of CD4^+^ T cells, CD8^+^ T cells, and dendritic cells, and CD40L on iNKT cells, promoted communication between each other. Hepatocytes release IL-7 to activate NKT cells; iNKT cells secrete TNF-α with dual functions on hepatocytes; iNKT cells express FasL and induce hepatocytes to express Fas, resulting in hepatocyte apoptosis. iNKT cells interact with CD8^+^ and CD4^+^ T cells through the secretion of IL-4 and IL-13, they have stimulatory effects on the main functional cells of CD4^+^ T cells, while on CD8^+^ T cells, they have both stimulatory and inhibitory effects, as iNKT cells can interfere with their chemotaxis. Tregs cells have a negative impact on CD8^+^ T and CD4^+^ T cells. Kupffer cells express LFA-1 and SIRPa, which bind to ICAM-1 and CD47, respectively, to activate iNKT cells. Kupffer cells secrete IL-12, IL-1b, IL-15, and AIM to recruit and promote iNKT cells. In return, iNKT cells produce pro-inflammatory IL-4 and IFN-g to act on Kupffer cells. Dendritic cells secrete IL-27 and IL-12 to activate NKT cells. Treg, regulatory T cells; DC, dendritic cells; IL, interleukin; TNF, tumor necrosis factor; IFN, interferon; SIRP, signal regulatory protein; LFA, lymphocyte function-associated antigen; ICAM, intercellular adhesion molecule; AIM, apoptosis inhibitor expressed by macrophages; TGF, tumor growth factor. This Figure is drawn using the BioRender website.

The interaction between NKT cells and immune cells is intricate and multifaceted. The interplay between iNKT cells and other immune cells as well as hepatocytes has been relatively elucidated in current research. iNKT cells are activated by lipid antigens through CD1d molecules which, in the liver, are expressed on T cells, macrophages, dendritic cells, and hepatocytes. The engagement of CD1d-TCR activate iNKT, and iNKT cells initiate TNF-α release, to either promote hepatocyte death or stimulate their regeneration (Aswad et al., 2005). iNKT cells secrete TNF-α and release death signals to hepatocytes through the FasL pathway, simultaneously secrete perforin and granzymes to promote liver cell injury (Santodomingo-Garzon and Swain, 2011). Meanwhile, IL-7 derived from hepatocytes also plays a crucial role in the maintenance of iNKT cells (Liang et al., 2012). The interaction between iNKT cells and CD8^+^ T cells has yielded conflicting experimental results. Some suggest that iNKT cells promote the cytotoxic activity of CD8^+^ T cells through the CD40/CD40L signaling pathway and secretion of cytokines such as IL-4 and IL-13 (Hermans et al., 2003; Dondji et al., 2008). However, activated CD8^+^ T cells secrete IFN-γ, which promotes the production of IL-4 and IL-13 by iNKT cells, and in turn, iNKT cells suppress the activity of CD8^+^ T cells by disrupting their chemotaxis to CCL5. The impact of iNKT cells on CD4^+^ T cells is mainly positive, leading to enhanced secretion of IFN-γ (Dondji et al., 2008; Shin et al., 2010). Dendritic cells are also believed to be involved in iNKT/T cell crosstalk, promoting the activation of both CD4^+^ and CD8^+^ T cells (Hermans et al., 2003). In addition, iNKT cell-induced Treg activation plays a crucial role in the depletion of CD4^+^ T cells and indirectly inhibits CD8^+^ T cells (Kim et al., 2006; Goubier et al., 2013). As an important antigen-presenting cell, the interaction between macrophages and iNKT cells is relatively complex, as it involves multiple surface and secreted molecules. One key mechanism of the crosstalk between macrophages and iNKT cells is through the LFA-1/ICAM-1 pathway. LFA-1 (lymphocyte function-associated antigen 1) and ICAM-1 (intercellular adhesion molecule 1) are located on the surface of macrophages and NKT cells, respectively, and have a high affinity for each other (Duwaerts et al., 2013). The binding of signal-regulatory protein alpha (SIRPa) on macrophages to CD47 on NKT cells also enhances the function of NKT cells (Beattie et al., 2010). In addition, IL-12, IL-1b, and IL-15 derived from Kupffer cells are believed to recruit iNKT cells, promote their activation, and participate in their maintenance (Golden-Mason et al., 2004; Tang et al., 2013; Cui et al., 2015; Hou et al., 2017). AIM (apoptosis inhibitor expressed by macrophages, also known as CD5L), a protein that is typically thought to inhibit apoptosis of CD4^+^CD8^+^ double-positive thymocytes, is secreted by Kupffer cells to protect iNKT cells from apoptosis (Kuwata et al., 2003). On the other hand, iNKT cells can produce large amounts of pro-inflammatory cytokines IL-4 and IFN-γ to enhance the function of Kupffer cells (Robert-Gangneux et al., 2012). Dendritic cells can secrete cytokines, including IL-27 (Wei et al., 2013) and IL-12 (Subleski et al., 2011), both of which have a positive impact on iNKT cells. In addition, iNKT cells also act as activators of dendritic cells by expressing CD40L. CD40L binds to CD40 on the surface of dendritic cells, forming an interaction (Bendelac et al., 2007; Subleski et al., 2011) (Figure 1).

## 5 The role of NKT cells in liver ischemia–reperfusion injury

An abrupt cessation of blood supply to a specific organ results in a condition of profound regional oxygen deprivation (Zimmerman et al., 2013). Reperfusion of blood flow following a period of acute cessation can lead to considerable cellular injury and subsequent impairment of organ function. This condition is known as ischemia-reperfusion injury (IRI) (Fahrner et al., 2016). IRI can trigger an inflammatory response in the early stage after transplantation, that would be leading to a significant dysfunction and failure of the transplanted organ if inadequately treated (Zimmerman et al., 2017). IRI is a multifaceted inflammatory process. Studies in preclinical models demonstrated that liver IRI is a localized inflammatory response that is mediated by both innate and adaptive immune cells. This inflammatory response is marked by the activation of Kupffer cells, the production of cytokines and chemokines by neutrophils, and the infiltration of lymphocytes and monocytes (Zhai et al., 2011; Zhai et al., 2013).

Recent findings indicate that NKT cells play an important role in causing hepatocellular damage in IRI (Zimmerman et al., 2017). Early observations by Shimamura et al. (2005) noted that the liver experiences a rapid expansion in the proportion of iNKT cells after undergoing selective portal vein clamping and reperfusion injury. After reperfusion, the number of iNKT cells in the liver gradually increases, reaching its peak at approximately 12 h, while the proportion of NK cells and other cells remains relatively stable. At the same time, alanine aminotransferase (i.e., ALT) also gradually increases, reaching its peak at approximately 10 h, following a similar trend as iNKT cells. However, when NKT cells are deficient in CD1d (−/−) mice, the extent of liver damage is significantly reduced (Shimamura et al., 2005). In a partial warm ischemia model, after being activated in a CD1d-dependent manner, iNKT cells, but not NK cells, contributes to hepatic IRI (*75*). Antibody depletion of iNKT cells, or both iNKT and NK cells, significantly reduced liver injury. It is noteworthy that iNKT cells start to produce IFN-γ 2 h after reperfusion (Lappas et al., 2006), suggests that iNKT cells participate in lymphocyte recruitment during the early post-perfusion period. In certain experiments, it was observed that mice treated with a synthetic adenosine 2A receptor (A_2A_R) agonist showed a significant 58% decrease in serum ALT levels (Day et al., 2004). The mechanism behind this reduction involves the direct inhibition of IFN-γ production by activated iNKT cells through the A_2A_R agonist. The group’s previous findings indicated a significant decrease in both IRI and transcript levels of proinflammatory cytokines/chemokines after administering a systemic A_2A_R agonist treatment (Day et al., 2004). Cao et al. (2009) presented a fascinating series of findings in a mouse model of partial warm ischemia (achieved through 60 min of portal vein occlusion followed by 6 h of reperfusion). The authors employed a focused approach of biochemical NKT activation prior to inducing ischemia in an effort to “precondition” the liver and enhance tolerance to IRI. NKT cells were preactivated through the intraperitoneal administration of the α-GalCer 60 min before hepatic ischemia. This preactivation led to a notable decrease in serum hepatic enzyme levels. The authors propose that these effects may be attributed to the expression of IL-13 and adenosine receptors. These findings hold great potential for the development of treatment and prevention strategies aimed at mitigating the damage caused by IRI in LTx.

Unlike activated iNKT cells, dNKT cells exert an anti-inflammatory effect, thereby restricting tissue damage (Bandyopadhyay et al., 2016). iNKT cells directly mediate hepatic IRI as mice lacking these cells are protected from injury in the setting of normal dNKT cells levels as early as 6 h post-reperfusion (reduction in serum ALT) (Cao et al., 2009). After 24 h of perfusion, the levels of tissue necrosis were significantly lower in NKT^−/−^ mice. During the study of sulfatide or LPC-mediated activation of dNKT cells in mice, researchers discovered that there is a rapid accumulation of iNKT cells in the liver, which is dependent on IL-12 and MIP2. However, these cells were found to be anergized, and as a result, mice that were treated showed protection against IRI. Anergy induction in iNKT cells induced following lipid-mediated activation of dNKT cells is different than that following chronic administration of type I ligand, α-Galcer (Parekh et al., 2005). Thus α-Galcer—but not sulfatide- or microbial-mediated anergy in iNKT cells require programmed death-1 (PD-1)/PD ligand (PDL)-1 signaling (Chang et al., 2008; Parekh et al., 2009). Another notable difference is that iNKT cells are activated before anergy induction with α-Galcer, a ligand that specifically binds to them. However, sulfatide, which is not a ligand for iNKT cells, does not activate them. dNKT cells-mediated inactivation of iNKT cells ultimately leads to a notable reduction in pro-inflammatory macrophages and neutrophil buildup, resulting in the inhibition of IRI (Arrenberg et al., 2011). Therefore, the mutual regulation and interaction between iNKT cells and dNKT cells are crucial for their functional regulation in diseases.

So far, reports barely demonstrated the role of NK-like T cells in IRI, but reasonable speculations can be made based on their characteristics, since the NK receptors expressing T cell population exhibits innate-like features. When exposed to IL-2 and IL-15 in IRI, both NK-like T cells and NK cells are capable of producing more IFN-γ than regular CD8^+^ T cells (Takayama et al., 2003; Guia et al., 2008; Gruenbacher et al., 2014). IL-15-induced CD8^+^CD56^+^ T cells exhibit a resident memory phenotype and an activated phenotype, expressing CD45RO and CD69 (Thulesen et al., 2001) and producing perforin and granzyme B (Guia et al., 2008; Correia et al., 2011; Poonia and Pauza, 2014) in IRI, which exhibit cytotoxic activity and may exacerbate the damage to hepatocellular.

## 6 The role of NKT cells in rejection and tolerance in LTx

Acute rejection is the primary reason for early graft failure and a significant risk factor for the long-term survival of LTx recipients. While the use of immunosuppressive agents such as calcineurin inhibitors (CNIs), rapamycin inhibitors (mTOR), or steroids after LTx significantly reduces rejection rates and improves survival rates, long-term use of these medications can lead to various complications, such as kidney injury, malignancies, and metabolic syndrome. These complications negatively impact the long-term survival of liver transplant recipients (Zhai et al., 2013; Huang H. et al., 2018). Therefore, the main aim of immunotherapy post LTx is to establish immune tolerance. Rejection is mediated by cells of the innate and adaptive immune systems, which recognize the allograft as non-self (Le Moine et al., 2002; Clayberger, 2009). Despite being a minor subset of T cells, NKT cells play a crucial role in regulating the immune system by bridging innate and adaptive immune responses (Kratzmeier et al., 2023).

During acute graft rejection, mononuclear cells infiltrate the portal tract and the accumulation of activated lymphocytes leads to the secretion of cytokines and chemokines, and subsequently causes liver tissue injury (Obara et al., 2005). The main antigens responsible for rejection are major histocompatibility complex (MHC) (Ronca et al., 2020). Damaged liver cells release damage-associated molecular patterns (DAMPs): HMGB1, free fatty acids, and heat shock proteins. This activates Kupffer cells via toll like receptors, stimulating release of pro-inflammatory cytokines such as IL-1, TNF, IFN and IL-12 (Dar et al., 2019). And these pro-inflammatory cytokines further activate other lymphocytes, exacerbating liver cell damage. Among them, activation of iNKT cells can be achieved through various means, including lipid antigens (especially α-Galcer) (Kobayashi et al., 1995; Osman et al., 2000; Shimizu et al., 2010), cytokines (such as IL-2 and IL-18) (Leite-De-Moraes et al., 2001; Uchida et al., 2007; Lind et al., 2009) and chemokines (including CXCR6) (Mossanen et al., 2019). Dong et al. (2007) reported that injured hepatocytes exhibit increased levels of CD1d expression in the context of liver transplantation rejection. Additionally, CD1d is expressed on antigen-presenting cells within the liver (Lappas et al., 2006; Kuboki et al., 2009). Under these circumstances, there is a significant recruitment and activation of iNKT cells, which initiate the production of various cytokines, including Th-1 (such as IFN-γ, IL-2, and TGF-β) and Th-2 (such as IL-4 and IL-10), as well as CD107a (Fernandez et al., 2012) and other molecules. Furthermore, iNKT cells, once activated, can induce hepatocyte apoptosis through multiple mechanisms, including upregulation of FAS antigen ligand (FASL), as well as secretion of cytotoxic molecules such as perforins and granzyme B (Takeda et al., 2000; Santodomingo-Garzon and Swain, 2011). As a result of these factors, there is an escalation of liver cell injury, along with damage to portal vein endothelial cells and bile duct epithelial cells. This, in turn, triggers a greater influx of inflammatory cells, thereby intensifying the rejection response. However, during the phase of severe acute rejection, there is a significant decrease of NK-like T cells compared to the control liver tissue and the less severe rejection phase (Werner et al., 2011). The author posits that this phenomenon may be attributed to the NKT cell overstimulation, which triggers decreased proliferation capacity and heightened FAS expression of NKT cells, as heightened levels of FASL resulted in the induction of apoptosis in iNKT cells (Takeda et al., 2000).

It was previously believed by many researchers that iNKT cells only promoted rejection, but this is now considered inaccurate. iNKT2 cells and iNKT10 cells are now recognized for their immunosuppressive and immunomodulatory effects, which can actually promote graft tolerance. Based on a rat rejection model, Liu et al. (2012) demonstrated that α-Galcer caudal vein injection substantially elevated the mRNA levels of the Th2-related cytokine, suggesting the iNKT may promote graft tolerance by regulating Th1/Th2 balance. Additionally, they also observed that the quantity of NK cells was notably reduced in liver grafts compared to the normal liver tissue. Conversely, the quantity of T cells in liver grafts was significantly higher than in the control tissue. The rise in T cells and the decline in NK and iNKT cells indicate a transition from innate to adaptive hepatic immunity within the liver graft (Werner et al., 2011). Although the precise mechanism remains elusive, it appears that the subpopulation ratio of NKT cells changes over time post-transplantation, primarily producing anti-inflammatory cytokines, thereby enhancing graft survival by increasing levels of IL-10, IL-4, and Treg cells (Jukes et al., 2007; Di Pietro and Falcone, 2014). Despite the fact that certain subgroups of iNKT cells exhibit contrasting roles and lack a definitive marker for their distinction, the intricate and diverse functionalities of iNKT cells have become more elucidated than ever before.

Throughout the years, it has been widely accepted that dNKT cells exert an inhibitory effect on inflammation, partly through engaging in cross-regulation and interaction with iNKT cells (Ware and Kumar, 2017). In the body, sulfatide-mediated stimulation of dNKT cells primarily activates hepatic plasmacytoid dendritic cells (pDCs) rather than conventional dendritic cells (cDCs), ultimately inducing tolerance in hepatic iNKT cells. This unique immunoregulatory pathway involves not only cross-regulation of iNKT cells but also the tolerization of hepatic cDCs, suppressing pathogenic Th1/Th17 cells (Kumar, 2013; Marrero et al., 2015; Bandyopadhyay et al., 2016; Dasgupta and Kumar, 2016; Dhodapkar and Kumar, 2017).

The T cells expressing NK receptors can cause organ rejection by attacking non-self targets as their conventional counterparts, but they are mainly believed to be associated with tolerance. During severe rejection in LTx, NK-like T cells are significantly reduced compared to conventional T cells, indicating the crucial role of these cells in maintaining the tolerogenic environment of the liver (Hu et al., 2021). In the recipient’s circulation, the number of donor-derived CD56^+^ T cells within 2 weeks after LTx positively corelated with the induction of graft tolerance (Moroso et al., 2010). The presence of T cells expressing NK cell receptors in immunotolerant liver raises the probability that they could either a represent of T cell tolerance or a suppressive population to control and maintain the aforementioned tolerogenic microenvironment (Doherty, 2016).

## 7 The role of NKT cells in graft infection after LTx

Due to the use of immunosuppressive agents after LTx, the graft is more susceptible to invasion by pathogenic microorganisms after surgery. The liver filters roughly one-third of the entire blood volume per minute (Racanelli and Rehermann, 2006; McNamara and Cockburn, 2016). Upon entering the liver, the blood flows at a slower rate through the sinusoids, a complex network of capillary-like vessels. This reduced flow rate enhances the ability of pathogens to detect and respond to the unique immune environment within the liver (Huang W. et al., 2018). Sinusoid-lining endothelial cells exhibit a high expression of CD1d, while Kupffer cells, as well as the hepatic and circulating dendritic cells, display a comparatively lower level of CD1d expression (Geissmann et al., 2005). Therefore, hepatic NKT cells within the blood vessels have easy access to CD1d-expressing cells at all times. The patrolling iNKT cells can be halted on the sinusoids in response to various stimuli, such as CD1d, inflammatory cytokines like IL-12 and IL-18 (Geissmann et al., 2005; Velázquez et al., 2008; Lee et al., 2010).

Hepatitis B Virus (HBV), Hepatitis C Virus (HCV), Epstein-Barr virus (EBV), and Cytomegalovirus (CMV) infections, as well as microbial antigens entering the liver from the gut through the portal veins, are the prevailing causes of graft infection following LTx (Lu et al., 2013; Narkewicz et al., 2013; Ware and Kumar, 2017; Rauber et al., 2019). CD8^+^ T lymphocytes serve as a pivotal element of the adaptive immune system, playing a vital role in the eradication of intracellular pathogens (Wong and Pamer, 2003; Raskov et al., 2021). Similar to CD4^+^ T cells, iNKT cells also exert a significant influence on the efficacy of CD8^+^ T-cell mediated immunity. Dendritic cells (DCs) are the major antigen-presenting cells (APCs) responsible for priming T cells during infection. More and more studies have shown that iNKT cells are another subset of DC licensing (Bousso and Albert, 2010; Gottschalk et al., 2015). DCs and iNKT cells release TNF-α and IFN-γ, respectively, which contribute to the activation of DCs through the CD40–CD40L pathway. This activation process enhances the ability of DCs to stimulate CD8^+^ T-cell responses by interacting with costimulatory molecules and inflammatory signals (Kitamura et al., 1999; Fujii et al., 2003). Upon priming, iNKT cells will accumulate in the marginal zone of the spleen and interact with CD8α^+^ conventional DCs (CD8α^+^cDCs) that express the chemokine receptor XCR1. These CD8α^+^cDCs are principal APCs responsible for cross-priming to active the immune response (Dorner et al., 2009; Bachem et al., 2012). Cross-priming allows DCs to cross-present extracellular antigens to CD8^+^ T cells, enabling them to acquire effector functions to defend against intracellular pathogens. This process is referred to as the “licensing” of DCs during infection (Qin et al., 2022). After initial activation, CD8^+^ T cells attract and recruit various cell types to the site where the antigen is recognized, in order to establish an ideal microenvironment for priming. pDCs play a critical role in antiviral infections by producing type I interferon (IFN) (Fitzgerald-Bocarsly et al., 2008). Shimizu K suggested that iNKT cell-mediated licensing of DCs for cross-priming of CD8^+^ T cells involves cross-talk between pDCs and XCR1^+^ DCs (Shimizu et al., 2013). The researcher further pointed out in subsequent studies that the expression of CXCR3 and CCR4 on CD8^+^ T cells mediated by iNKT cells may affect the fate of CD8^+^ T cells (Valente et al., 2019). Direct interaction between TCRs on iNKT cells and the transient upregulated CD1d molecules on activated CD8^+^ T cells enhanced IFN-γ production and the proliferation and cytotoxicity of CD8^+^ T cells in a dendritic cell-independent manner to eradicate pathogens (Qin et al., 2019).

During HBV and HCV infections, different subsets of iNKT cells also exert distinct functions. Both mouse and human iNKT cells have the ability to identify lipid and glycolipid antigens derived from either self or microbial sources, which are presented on MHC class-I-like CD1d molecules (Kawano et al., 1999; Bendelac et al., 2007). The elevated levels of CD1d on bile cells infected with HBV and HCV enhance the activation of iNKT cells (Durante-Mangoni et al., 2004). Activated iNKT1 cells mediate type 1 immune response by producing IFN-γ, as well as perforin and granzymes, leading to liver damage (Kaneko et al., 2000). Furthermore, iNKT1 cells facilitate the activation of cytotoxic T lymphocytes (CTLs) and thus contribute to the inhibition of HBV (Ito et al., 2008). After activation, iNKT2 cells produce a large amount of IL-4 and IL-13 to mediate type 2 immune responses. Type 2 immunity and its effector cytokines IL-13 and IL-4 play various roles in the immune system, such as promoting tissue repair in a local immune response and regulating type 1 immunity (Gause et al., 2020). iNKT10 cells have a regulatory effect on immune responses through the production of IL-10. This can occur either through direct secretion of IL-10 or by influencing other IL-10 producing cells, such as Treg cells. Raus S has developed an HCV related rodent hepatitis virus (NrHV), which was isolated from Norwegian rats (Raus et al., 2022). They found that in the models of NrHV infection in CD1d^−/−^ mice with iNKT deficiency and normal mice, the levels of ALT were significantly increased in the iNKT-deficient mice after infection. The elevated levels of ALT indicate heightened liver damage in the absence of iNKT cells, indicating a potential immune-regulatory and/or tissue protective function of these cells, possibly through the secretion of type 2 cytokines. Accordingly, iNKT cells that are biased towards type 2 immunity play a limiting role in acute liver injury during infection. This is achieved through direct or indirect means, including the regulation of antiviral effector functions of T cells that are specific to the virus (Raus et al., 2022). However, their effect on hepatic cells is dual in nature and could be attributed to the distinct roles played by their subgroups. Certain research indicates that during particular immune response mechanisms, diverse immune microenvironments within the liver shape the function and polarization of iNKT cells (Raus et al., 2022).

CMV stands as the prevailing infection in the post-transplant environment, presenting the greatest susceptibility during the initial 3 months following transplantation, particularly among patients with heightened immunosuppressive states (Razonable et al., 2004). Following liver transplantation, the immunosuppressive treatment hampers the activity of memory T cells, resulting in approximately 10% of patients encountering B cell proliferation induced by the EBV. EBV-infected B cells typically remain dormant without causing symptoms, but in rare cases, they may experience uncontrolled growth and transform into a clonal population. This can lead to the development of post-transplant lymphoproliferative disorder (PTLD), which occurs in approximately 4.7% of pediatric liver transplant recipients and 1% of adult recipients (Burra et al., 2006; Abu-Elmagd et al., 2009; Kamdar et al., 2011). The infection of the liver graft by cytomegalovirus enhances the resilience of the graft against immune rejection by inducing the production of type I interferon (Hokeness-Antonelli et al., 2007) and attracting Th1 cells that secrete IFN-γ (Bruns et al., 2015), a crucial factor for achieving liver transplant tolerance in animal studies (Mele et al., 2003). What is even more intriguing is that in the study conducted by Xiao W, it was that after infection with the EB virus, iNKT cells produced a skew towards Th1 cytokines, which appears to be contrary to the effects of HBV and HCV (Xiao et al., 2009). The identification of EB virus antigens and the mechanisms by which they influence the cytokine and immune response patterns within cells are still subject to further investigation. The integration of weak TCR signals and IL-12R-mediated signals is a potential factor leading to the production of Th1-biased cytokines by iNKT cells (Brigl et al., 2003).

Typically, dNKT cells are deemed to possess anti-inflammatory properties and modulate the activity of iNKT cells (Terabe and Berzofsky, 2007; Subleski et al., 2011). Out of all the phospholipids, LPC has been demonstrated to be acknowledged by human and murine dNKT cells, while murine iNKT cells do not recognize it (Gapin et al., 2013; Maricic et al., 2014). Given that inflammation can lead to changes in endogenous lysophospholipid levels through the hydrolysis of phospholipids (Knowlden and Georas, 2014), it is possible that dNKT cells reactive to lysophospholipids could be involved in regulating liver inflammation. However, some reports have also indicated the role of dNKT cells in promoting chronic inflammation (Weng et al., 2017). Activation of dNKT cells using endogenous glycolipid sulfatide suppresses the inflammatory reactions triggered by CD4^+^ T (Arrenberg et al., 2011) and iNKT cells (Halder et al., 2007) in mice. In a rat model, stimulation of dNKT cells with SCP2 peptide induces an inflammatory response by promoting the secretion of pro-inflammatory cytokines IL-5 and IL-6 (Iinuma et al., 2015). The role of dNKT cells in hepatitis viral infection is a topic of debate. However, in a mouse model of ConA-induced hepatitis, the activation of dNKT cells with sulfatide or LPC induces anergy in iNKT cells, which in turn suppresses inflammation-induced liver damage (Maricic et al., 2014). On the other hand, hepatic dNKT cells facilitate the progression of liver damage in a transgenic mouse model of acute HBV infection (Baron et al., 2002). Zeissig had unveiled the protective function of type II NKT cells in a murine model of hepatitis B by employing adenoviral particles expressing HBV (Zeissig et al., 2012). Activation of dNKT cells induces the activation of conventional T cells and the production of pro-inflammatory cytokines, leading to the exacerbation of liver damage in mouse models of autoimmune hepatitis (Weng et al., 2017). The cumulative findings of these investigations demonstrate the ability of type dNKT cells to exert influence on the physiology of various viral and bacterial infections, wherein they can either facilitate the development of beneficial innate and adaptive immune reactions or contribute to the infliction of tissue damage caused by pathogens.

CD56 expressing T cells expand in viral infection and exert anti-viral effects (Koh et al., 2023). Under conditions of inflammation, these T cell displayed restricted TCR diversity and are capable of amounting immune response in a NK-related receptor regulated bystander manner, that is especially well documented in HAV infection (Kim et al., 2018). When expose to IL-15 and IL-18, the well characterized cytokines contribute to bystander activation, the NK-like T cells produce higher levels of IFN-γ than conventional CD8^+^ T cells (Takayama et al., 2003; Guia et al., 2008; Gruenbacher et al., 2014). In addition to IFN-γ, upon activation, NK-like T cells secrete elevated levels of TNF-α, IL-5, and IL-13 (von Geldern et al., 2006), and exhibit robust production of perforin and granzyme B (Guia et al., 2008; Correia et al., 2011; Poonia and Pauza, 2014). As for the HDV infection, the NKp30 and NKG2D expression on intrahepatic resident memory T cells positively correlated with the liver enzyme activity, and they act cytotoxic activity in a beyond HDV-specific NKG2D-dependent manner (Kefalakes et al., 2021). Patients infected with HBV have been found to have increased levels of circulating NK-like T cells, as well as IL-17, which is associated with HBV mRNA levels that decrease as the patient recovers (Diao et al., 2014). In addition, it was observed that CD16^+^ NK-like T cells exhibited a notable elevation in the bloodstream of individuals infected with Hepatitis E virus (HEV), and those who had recovered displayed augmented levels of the cytotoxic receptors NKp44 and NKp46, as well as the activating receptor NKG2D (Srivastava et al., 2008; Das and Tripathy, 2014). June-Young K. and others summarized that a unique population of CD56^hi^CD161^−^CD8^+^T cells was identified in the liver, which exhibited NK-like T cells activation through NKG2C ligation independent of TCR (Koh et al., 2022). The researchers discovered that this unique population was notably increased in the livers of individuals suffering from chronic HBV infection, indicating its potential pathogenic significance. In CMV-seropositive patients, the NKG2C expressing CD8^+^ T cells display a narrow TCR Vβ-chain usage, indicating a clonal expansion of these cells (Sottile et al., 2021). The KIR^+^CD56^+^ T cells increased in CMV-infected patients after bone marrow transplantation, and these cells also lyses the CMVpp65-pulsed target cells in a KIR-dependent manner (Chan et al., 2013). Collectively, the NK-like T cells, by expressing the innate receptors, functions in a TCR-independent and NK receptor-dependent way to participant the virus infection after liver transplantation.

## 8 Prospects of NKT cells in LTx immunotherapy

Currently, there is a growing body of literature reporting on the increasing prominence of NKT cell-based immunotherapy in the context of cancer treatment. CAR-NKT cells can serve as a secure and efficacious platform, akin to CAR-T cells, for the advancement of tumor immunotherapy (Simon et al., 2018). The recognition of conserved lipid antigens presented via CD1d by iNKT cells, along with their capacity to coordinate the immune response against tumors through cytokine signaling, renders them highly appealing for the development of cancer vaccines (Nelson et al., 2021).

The application of NKT cell-related therapies in transplantation is still in its very early stages. Based on their functional characteristics, we can now envision potential therapeutic strategies that may be realized in the future. The use of bone marrow mesenchymal stem cells has been found to effectively prevent liver transplant rejection by enhancing the inhibitory signals directed towards NKT cells, thereby reducing the production of IFN-γ (Cao et al., 2021), while the acceptance of allografts is influenced by IL-10 (Chen et al., 2008). As mentioned earlier, both iNKT2 and iNKT10 subsets within the iNKT cell population, and dNKT cells, exert suppressive effects on inflammatory responses and promote tolerance. Increase the proportion or function of iNKT2 and iNKT10 cell subpopulations in the entire iNKT cell disrupt the balance between Th1 and Th2 responses leads to a transition to Th2 cell (Liu et al., 2012), that favor the tolerance induction. While the exact mechanism remains unclear, future strategies for NKT cell-based transplantation immunotherapy would focus on inducing the release of the immunosuppressive cytokines by influence the T cell subsets balance by iNKT cells.

## 9 Limitations of current research

Based on current findings, iNKT cell are functional heterogeneous, that may also undergo dynamic changes. The apparent contradictions in some of their functions can be attributed to the distinct functionalities of their various subgroups, but the classification of NKT subpopulation is not as clear as their conventional T cell counterpart. Although five subgroups of iNKT cells have been identified in mouse experiments, there is currently a lack of specific markers to distinguish subgroups of human iNKT and dNKT cells, as well as a lack of unified classification criteria. This is partly attributing to their rarity and the tissue specific distribution, obtaining sufficient human NKT cell samples for research, particularly from the human liver, is challenging. This has posed immense challenges in the profound exploration of iNKT cells, and has further hander their clinical application by the uncertainty of the immune-boosting or immune-regulating properties of NKT subsets under distinct condition.

The expression of NK receptors is observed in a multitude of T cells instead of in a uniformed subset. Expression of the NK-related receptors may suggest an altered functional regulation pattern and activation manner, and also reflect T cell differentiation stage and associate with T cell aging. However, the current research lacks a comprehensive and unambiguous classification of these cells, and the functions of their subgroups have not been systematically elucidated, and the blurred boundary between NK-like T cell and NKT cells in some study further complicate this issue.

## 10 Conclusion

As previously mentioned, NKT cells act as a bridge between innate and adaptive immunity, and are important factor in the context of liver transplant ischemia-reperfusion injury, graft rejection and tolerance, and transplant infections. There are also interactions between different subgroups of NKT cells, which collectively regulate the human immune response. In general, with the in-depth research in recent years, iNKT cells are not a single functional cell, but a population of cells with diverse subgroups. Among them, iNKT1 cells mainly have pro-inflammatory functions and can exacerbate liver cell damage in conditions such as ischemia-reperfusion injury, rejection reactions, and infections. On the contrary, iNKT2 cells and iNKT10 cells secrete cytokines such as IL-4, IL-13, and IL-10, respectively. These cytokines are considered to have immunosuppressive and immunomodulatory effects, which can promote transplant tolerance. dNKT cells have been shown to inhibit the response of iNKT cells, and their main function is to suppress and regulate immune responses. Some studies suggest that dNKT cells can also be divided into pro-inflammatory and anti-inflammatory subgroups, but the specific functions and classification methods are currently unclear. Similarly, NK-like T cells also tend to have pro-inflammatory effects similar to iNKT1 cells. It is believed that cell therapy targeting NKT cells can be applied to the clinic to improve the immune environment after LTx in the future.
